# N-Net: A novel dense fully convolutional neural network for thyroid nodule segmentation

**DOI:** 10.3389/fnins.2022.872601

**Published:** 2022-09-01

**Authors:** Xingqing Nie, Xiaogen Zhou, Tong Tong, Xingtao Lin, Luoyan Wang, Haonan Zheng, Jing Li, Ensheng Xue, Shun Chen, Meijuan Zheng, Cong Chen, Min Du

**Affiliations:** ^1^College of Physics and Information Engineering, Fuzhou University, Fuzhou, China; ^2^Fujian Key Lab of Medical Instrumentation and Pharmaceutical Technology, Fuzhou University, Fuzhou, China; ^3^Imperial Vision Technology, Fuzhou, China; ^4^Fujian Medical Ultrasound Research Institute, Fuzhou, China; ^5^Department of Ultrasound, Fujian Medical University Union Hospital, Fuzhou, China

**Keywords:** deep convolutional neural network, medical image segmentation, dilated convolution, multi-scale input layer, thyroid nodule

## Abstract

Medical image segmentation is an essential component of computer-aided diagnosis (CAD) systems. Thyroid nodule segmentation using ultrasound images is a necessary step for the early diagnosis of thyroid diseases. An encoder-decoder based deep convolutional neural network (DCNN), like U-Net architecture and its variants, has been extensively used to deal with medical image segmentation tasks. In this article, we propose a novel N-shape dense fully convolutional neural network for medical image segmentation, referred to as N-Net. The proposed framework is composed of three major components: a multi-scale input layer, an attention guidance module, and an innovative stackable dilated convolution (SDC) block. First, we apply the multi-scale input layer to construct an image pyramid, which achieves multi-level receiver field sizes and obtains rich feature representation. After that, the U-shape convolutional network is employed as the backbone structure. Moreover, we use the attention guidance module to filter the features before several skip connections, which can transfer structural information from previous feature maps to the following layers. This module can also remove noise and reduce the negative impact of the background. Finally, we propose a stackable dilated convolution (SDC) block, which is able to capture deep semantic features that may be lost in bilinear upsampling. We have evaluated the proposed N-Net framework on a thyroid nodule ultrasound image dataset (called the TNUI-2021 dataset) and the DDTI publicly available dataset. The experimental results show that our N-Net model outperforms several state-of-the-art methods in the thyroid nodule segmentation tasks.

## 1. Introduction

The thyroid gland is a butterfly-shaped endocrine gland, which lies in the anterior part of the neck just below the thyroid cartilage (Chang et al., 2010b). Generally, some thyroid nodules have a clear and regular boundary, but others are in blurred and irregular margins. Previous studies (Chen et al., 2020b) have shown that hypoechoic nodules with irregular margins are more likely to develop into malignant nodules. The detection rate of thyroid nodules in the general population is 3–7%, and the diagnostic rate with the help of high-resolution ultrasonography is 20–76%. Among the cases detected, 5–15% of patients with nodules are confirmed as having thyroid cancer (Brito et al., 2013). Although most thyroid nodules are benign (non-cancerous), a fraction of them tends to turn into thyroid cancer (Chen et al., 2020a). In order to diagnose and treat thyroid cancer at the earliest phase, it is desired to detect and diagnose the nodules as early as possible.

Ultrasound and computed tomography (CT) are the most widespread approaches for the diagnosis of thyroid diseases. In clinical practice, ultrasound examinations are dependent on visual inspection by experienced clinicians, which requires concentrated attention and a high level of skill. The diagnosis process is time-consuming, labor-intensive, and prone to operation bias. Furthermore, it is hard to identify the subtle differences between malignant and benign nodules. Geometry and margins of thyroid nodules are the key features to distinguishing between malignant and benign nodules (Lee et al., 2011). For example, taller-than-wide shape and spiculated boundary are two of the major characteristics to determine whether the thyroid nodules are malignant or not (Moon et al., 2008). Segmentation plays a critical role in the detection of nodules and the generation of a region of interest (ROI) for subsequent analysis in thyroid ultrasound images (Ma et al., 2017). Incorrect segmentation results will lead to misdiagnoses that are based on boundary features. Therefore, accurate thyroid nodule segmentation is essential to promote the study of thyroid disease diagnoses, providing valuable information for clinicians to make the best possible diagnostic judgment.

Many innovative methods have been proposed for accurate medical image segmentation. Traditional approaches include contour and shape based methods (Chang et al., 2010a), and region based methods (Poudel et al., 2018). In the study of Poudel et al. (2018), histogram equalization was utilized to adjust the contrast and a median filter was used to reduce speckle noise. In the end, the contour evolution was used to improve the segmentation of thyroid nodules in ultrasound images.

Methods based on unsupervised learning have been also successfully used in medical image segmentation. Tsantis et al. (2006) designed a hybrid unsupervised learning model for thyroid nodules segmentation in ultrasound images integrating wavelet-based edge detection and Hough transformation, which is able to outline thyroid nodules regardless of discontinuous boundaries and extensive structure noise. Zhao et al. (2013) proposed a thyroid nodule segmentation method based on the normalized cut of graph theory, which can reduce image noise by using homomorphic filtering.

The restriction of these methods is the use of hand-crafted features to obtain segmentation results. On the one hand, the selection of hand-crafted features may be biased to specific tasks, which hampers the generalization performance of the model. The extracted features may be well utilized on one type of dataset in a specific task but not as well on another type of image data set or in another segmentation task. On the other hand, it is difficult to extract representative low-level features for various applications. Therefore, there is no universal method to extract the hand-crafted features.

Over the past few decades, due to its ability to extract high-level semantic features from raw images, a deep convolutional neural network (DCNN) has been widely used in the field of medical image segmentation (Sevastopolsky, 2017; Yu et al., 2018; Zhang et al., 2019a). Without extracting hand-crafted features as in traditional methods, DCNN-based approaches can learn useful features for the segmentation tasks. A fully convolutional network (FCN) (Long et al., 2015) was first adopted to get accurate 2D segmentation results (Ma et al., 2017). Since the FCN has consecutive spatial pooling and convolution strides, the learned features have a significant loss in spatial details, which will affect the accuracy of segmentation. Ronneberger et al. (2015) proposed a U-shape FCN referred to as U-Net and it has become the mainstream in medical image segmentation (Roy et al., 2017; Norman et al., 2018; Sarker et al., 2018; Jha et al., 2020; Zhang et al., 2020; Isensee et al., 2021; Jin et al., 2021). The U-Net structure includes an encoder part and a decoder part. The purpose of the encoder is to reduce the spatial dimension of feature maps gradually and learn high-level semantic features. The Decoder aims to recover the object details and spatial dimensions gradually with the use of upsampling layers. Many variants of U-Net models have been designed for various medical image segmentation tasks. Oktay et al. (2018) designed U-shape DCNN coupled with a novel attention gate (called attention-U-Net) for pancreas segmentation in CT abdominal images. Fu et al. (2018) proposed a modified U-Net architecture (referred to as M-Net) for joint optic disc and cup segmentation. A multi-scale input layer and a hybrid loss were utilized in M-Net, which can provide deep supervision to improve the segmentation performance. Based on the multi-scale input layer and hybrid loss, Zhang et al. (2019b) proposed an attention guided network (AG-Net) for retinal image segmentation and Mehta and Sivaswamy (2017) presented a novel M-Net to segment brain structures from 3D Magnetic Resonance Images (MRI).

Inspired by U-Net, numerous studies have attempted to improve the structure of U-Net for various image recognition tasks. For example, Zhou et al. (2019) creatively proposed a simple and effective modified U-Net that redesigns the original U-Net skip connection. It reduces the loss of information in the copy aggregation from the encoder to the decoder. However, it has many parameters, and the information of the upper layer is not integrated, which causes the fine-grainedness of the Decoder part to be still not fine enough. Amer et al. (2020) proposed a new model based on U-net and incorporated with dilated convolution, where residual blocks are employed instead of the basic U-Net units. Each block is enriched with a squeeze and excitation unit for channel-wise attention and adaptive feature re-calibration. In Ibtehaz and Rahman (2020), Ibtehaz et al. designed MultiResUNet leading to two advantages. First, the MultiRes block is proposed and replaces all 2D convolution blocks in the classical U-Net. Second, a Res path with four 3 × 3 convolution and four short connections of Residual properties are creatively proposed to replace skip connections. Gudhe et al. (2021) proposed an innovative approach to segment lesions/tumors by exploiting the inherent properties of the residual learning and the dilated convolutions to efficiently capture both the local and the contextual features. Due to the great success of convolutional neural networks (such as U-Net) in the field of medical image segmentation, we chose U-Net as the basic framework when designing the network. However, U-Net based methods are a double-edged sword, because the intrinsic features of convolution operations result in limited receptive fields, and they fail to establish long-range dependencies and global contextual connections.

Therefore, we designed a stackable dilated convolution (SDC) block to solve the problem of restricted receptive fields. Meanwhile, we added the attention guidance (AG) module before the jump connection structure to establish long-range dependencies, and introduce a multi-scale mechanism to solve the problem of ignoring the global contextual information of different scales. In this study, we propose a combined fully convolutional network for thyroid nodule segmentation using ultrasound images, referred to as N-Net. The main contributions of this study are 3-fold as follows:

1) We propose an N-shape fully convolutional network, which contains a multi-scale U-shape convolutional network to learn multi-scale feature representations. The multi-scale input layer is used to construct an image pyramid input, which can achieve multi-level receiver field sizes, thus learning rich feature representations. This makes the network learn both the global and the local information of thyroid ultrasound images to extract the multi-level features.

2) An attention guidance module is proposed to preserve the structural semantic features to improve the segmentation performance. This module makes the model pay more attention to the thyroid regions of the input image and reduces the effect of mixed noise in ultrasound images on our network. It can be considered as semantic guidance in the network to acquire more precise semantic representations.

3) We designed a stackable dilated convolution (SDC) block to encode the high-level semantic contextual features from feature maps. The SDC block adopts a hybrid (both parallel and cascade) dilated convolution with different dilation rates. Our network can learn the semantic features which may be lost in bilinear upsampling through the SDC block.

The remainder of this article is organized as follows. Section 2 introduces related study. Section 3 describes the proposed method in detail. Section 4 presents the experiment results. Conclusions are summarized in Section 5.

## 2. Related study

### 2.1. Traditional segmentation methods

Traditional segmentation methods include contour and shape based methods and region based methods. Tsantis et al. used the morphological and wavelet-based features of nodules in thyroid ultrasound images to assess the malignancy risk of thyroid nodules on ultrasonography in 2009 (Tsantis et al., 2009). Liu et al. applied a level set-based method to the segmentation of cardiac MRI images (Liu et al., 2014) and glandular staining images (Wang et al., 2017). However, due to the low contrast, speckle echoes, blurred margins of thyroid nodules, and shadows of calcification points in the thyroid ultrasound images, the direct use of level set approaches cannot result in a good performance for segmenting thyroid nodules. Poudel et al. (2017) proposed a 3D thyroid segmentation method, which performed 2D image segmentation using the active contour method and then fused the 2D segmentations to get the final 3D results. Although traditional machine learning algorithms perform well in detection and segmentation, with the advent of the era of big data, the performance of traditional machine learning has become a bottleneck in fully utilizing big data.

### 2.2. Deep learning based segmentation methods

In recent years, models based on DCNN have demonstrated significant improvements IN thyroid nodule segmentation (Ma et al., 2017; Ying et al., 2018; Shen et al., 2020; Tang and Ma, 2020; Wang et al., 2020; Zhang et al., 2020). In Ying et al. (2018) proposed a method using cascaded U-Net and VGG-19 (Simonyan and Zisserman, 2014) network to segment the ROI area of thyroid nodules to assist doctors in diagnosis. However, the segmentation results of nodules with complicated edge details are not accurate enough. Moreover, Zhang et al. (2020) proposed a cascade U-Net to segment and classify thyroid nodules. The cascaded U-Net is composed of U-Net-I and U-Net-II, which segment the nodules in the image with uniform resolution and original resolution, respectively. In addition, this method takes segmentation as an auxiliary task to improve classification performance. Tang and Ma (2020) designed a robust coarse-to-fine two-stage segmentation algorithm, which is based on Deeplabv3+ (Chen et al., 2018) architecture. Shen et al. (2020) designed a two-stage network, using the detection and segmentation results to generate class-discriminative cues for improving the classification performance. This method won third place in the classification task at the TN-SCUI2020 challenge. As the first place in the segmentation task ranking in the TN-SCUI2020 challenge. Wang et al. (2020) proposed a cascaded segmentation framework and a resnet-based dual attention classification network to improve the segmentation and classification of thyroid nodules, respectively. In the future study, in order to be applied to assist doctors in the formulation of preoperative plans, the network can be designed to be lighter and faster while retaining most of the accuracy of the algorithm.

In pixel-wise semantic segmentation tasks, the segmentation result has the same size as that of the input image. Generally, a deep convolutional layer can learn useful feature representations, but max-pooling layers may lead to the loss of semantic and spatial information. Therefore, methods such as bilinear interpolation, deconvolution, and dilated convolution are used in CNN-based networks to solve this problem. When dilated convolution is adopted, the information in the down-sampled feature map can be more accurately decoded into the output label than that using the bilinear interpolation or the deconvolutional layer. The dilated convolution (or atrous convolution) was originally proposed for the efficient computation of the wavelet transform (Holschneider et al., 1990). Chen et al. (2017a) used dilated convolution for dense segmentation. The dilated convolution in the bottleneck is used in the DeepLab-type of models that introduced this idea in 2017 (Chen et al., 2017b). Since it uses a larger kernel than the standard convolutional layer, the dilated convolution can enlarge the size of the receptive field without increasing the number of parameters or the amount of computation. The main idea of dilated convolution is to insert a "hole" (zero) between the pixels of the convolution kernel to improve the resolution of the image, thus yielding dense feature extraction in DCNN.

## 3. The proposed method

**Figure 1:**
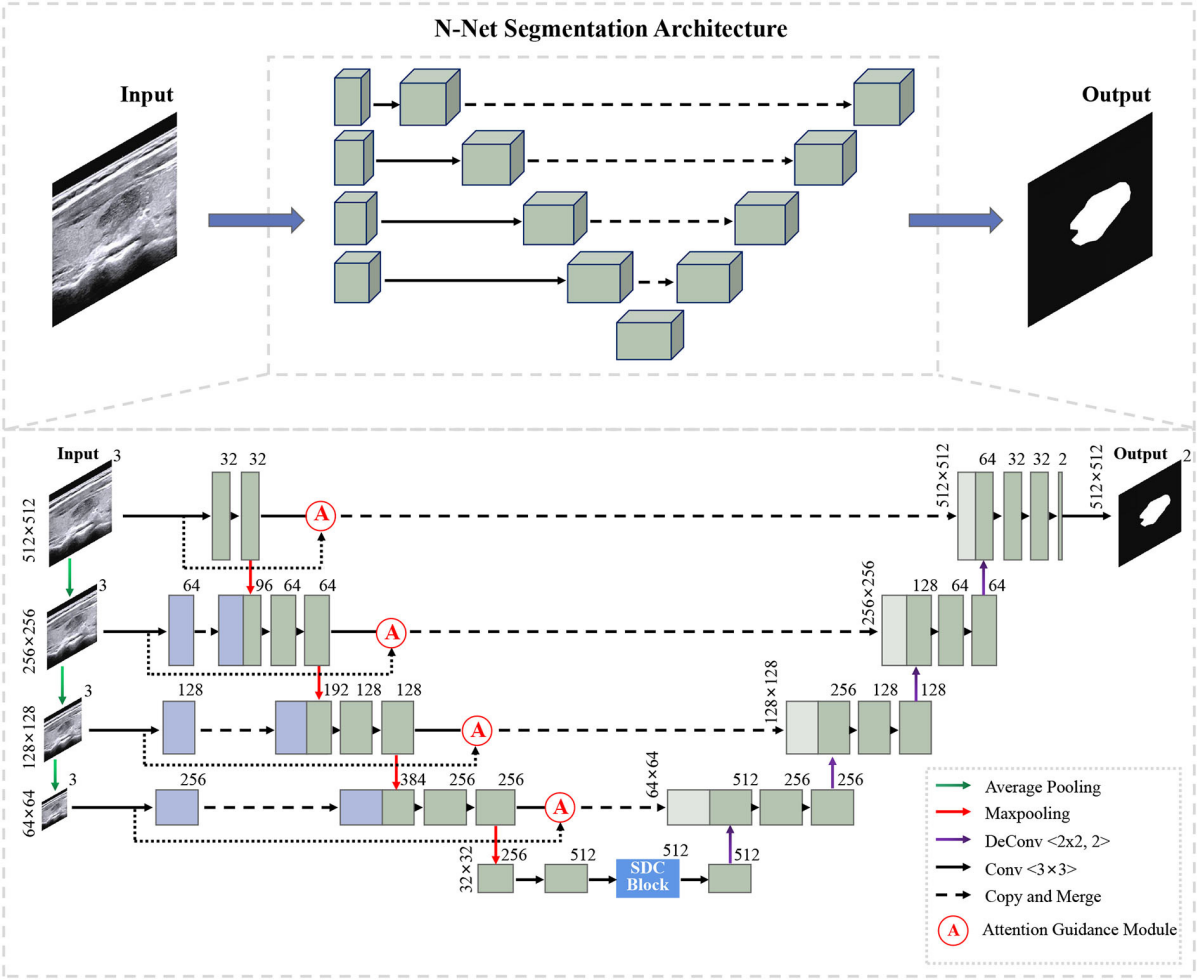
Illustration of our N-Net segmentation framework.

Figure 1 shows the overall structure of our N-Net. The upper part is the three-dimensional overall N-shape architecture of our network. Additionally, the detailed modules of the N-Net model are shown in the following section. The "dash arrow line" between the two blue blocks of the coding section represents data flow (the copy of the data). Our network is an N-shape encoder-decoder structure. It is composed of three parts. The first part is a multi-scale input layer that can build an input image pyramid and achieve a multi-level receptive field fusion, preserving both the low-level and the high-level features. The second part is a U-shape convolutional network, which is used as the backbone for learning high-level semantic feature representation. In this part, the attention guidance block (Oktay et al., 2018) is employed to guide the encoding layers. In this way, the N-Net can pay more attention to the foreground pixels in an input image for the segmentation task. In the third part, motivated by atrous convolution (Chen et al., 2017a), we designed a stackable dilated convolution (SDC) module to extract contextual semantic information and generate high-level feature maps. In the end, we optimized the segmentation network by minimizing a cross-entropy loss.

### 3.1. U-shape convolutional network

In our study, the proposed U-shape convolutional network is optimized based on the U-Net (Ronneberger et al., 2015) architecture, which has been demonstrated to be very effective and successful for medical image segmentation (Ronneberger et al., 2015; Dong et al., 2017; Norman et al., 2018). It consists of an encoder flow (left side) and a decoder flow (right side). In the encoding part, each step has a cascade of convolutional layers of size 3 × 3 and maxpooling by 2 × 2, which reduces the size of input by half and generates a series of feature maps. Moreover, the rectified linear unit (ReLU) activation function is followed, which introduces non-linearity into the model. The decoding part is very similar to the encoding part, with one exception: upsampling layers replace maxpooling to double the size of the feature maps and restore it to the original size of the input image. The skip connections transfer the corresponding feature maps from encoder flow and concatenate them to up-sampled decoder feature maps, which are introduced to enable the network to learn rich features. Meanwhile, skip connections can provide enough information to deduce the fine grain labeling of the image without any post-processing (Ronneberger et al., 2015).

### 3.2. Muti-scale input layer

We propose a multi-scale input layer to generate an input image pyramid, which contains four-layer input images with different resolutions. The strategy has been shown to improve the segmentation performance significantly in other tasks (Mehta and Sivaswamy, 2017; Fu et al., 2018). Some other studies (Li and Yu, 2016; Liu et al., 2017) exploited the multi-scale images to multi-branch models respectively and fused the feature maps in the last convolutional layer. Our N-net applies three 2 × 2 average pooling layers to downsample the input original image and build a pyramid input in the encoder part, avoiding the considerable growth of parameters and preserving the low-level feature representation. This layer can provide multi-level receiver field sizes and obtain rich feature representations.

### 3.3. Attention guidance module

**Figure 2:**
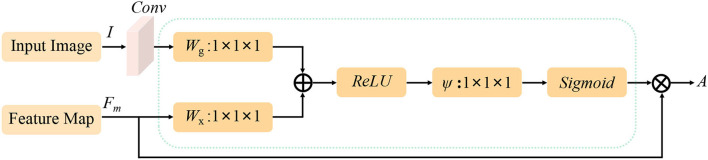
Flowchart of the attention guidance module. *I* and *F*_*m*_ are the inputs of the attention guidance module and *A* is the calculated attention map.

**Figure 3:**
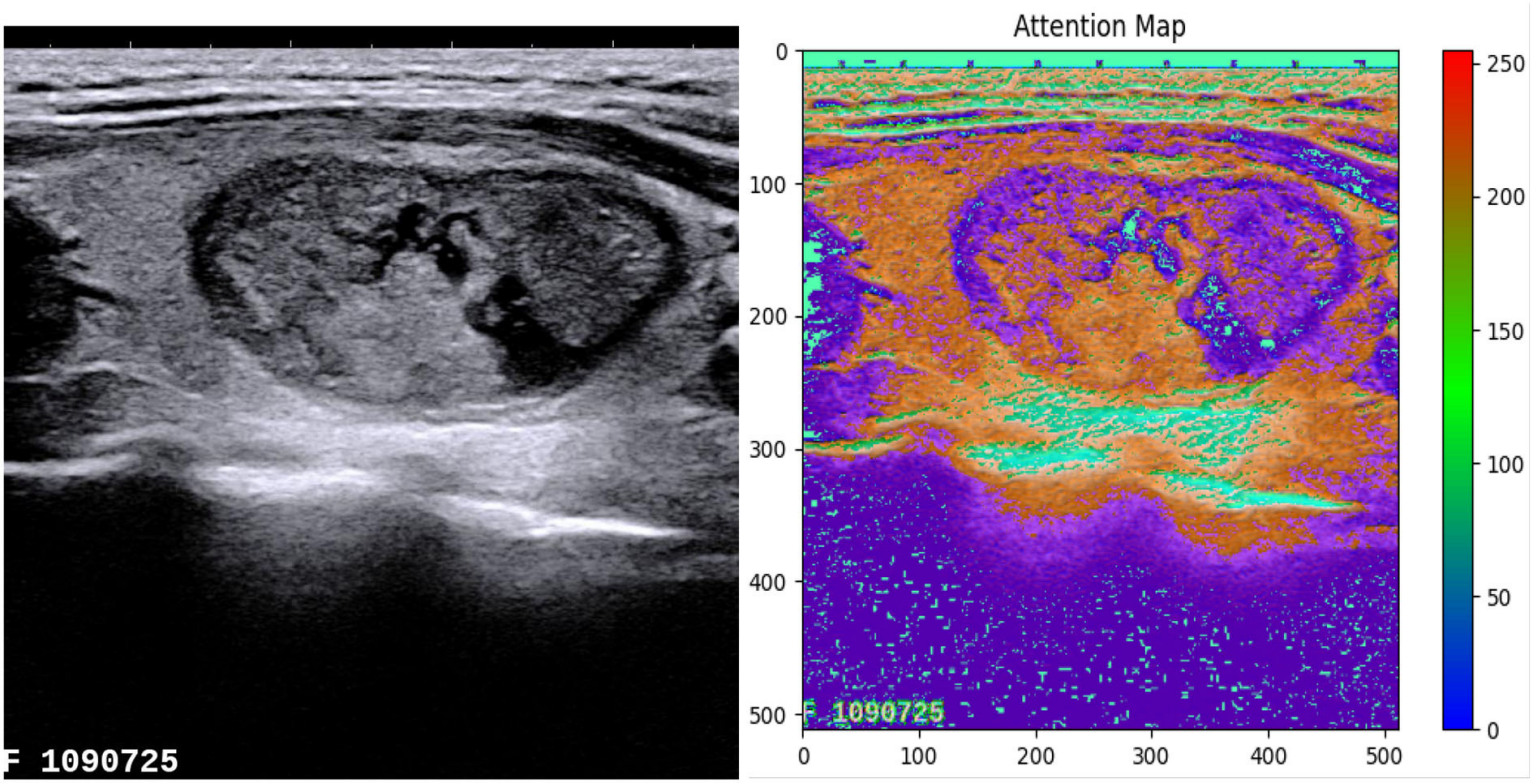
The attention map highlights the foreground region of the thyroid nodule.

We use the attention guidance module to improve the sensitivity of the network to the foreground and reduce the effect of the background noises. This module can also tackle the boundary blur problem caused by upsampling. In addition, it can learn high-level and semantic features, thus providing rich information for improving the segmentation. In contrast, the attention module proposed by Oktay et al. (2018) pays less attention to the irrelevant area in an input image while highlighting the regions useful for a specific task. As shown in Figure 2, our attention guidance module consists of the input image *I* and a feature map *F*_*m*_. A channel-wise 1 × 1 convolutional layer is used to perform a channel transformation. Two transformed feature maps are integrated with an element-wise coupled and a ReLU layer. A 1 × 1 convolutional layer is applied as an additional linear transformation with a sigmoid activation and feature map *F*_*m*_ to produce the final attention map *A*. We have compared the performance by adding the AG module before and after the skip connection and found that the former has a greater performance improvement for segmentation. In addition, since this module is placed before skip connections, it can be considered as semantic guidance, helping low-level features integrate high-level semantic representations while guiding the subsequent upsampling process through skip connections. As can be seen from the attention map in Figure 3, the attention guidance module makes the model pay more attention to the foreground region of the input image during the feature extraction process, which is the thyroid nodule region of the segmentation task.

### 3.4. Stackable dilated convolution block

We propose a stackable dilated convolution (SDC) block to encode the high-level semantic contextual features from feature maps. Compared with the standard convolutional layer, the dilated convolution uses a larger convolutional kernel and enlarges the receptive field without reducing the resolution of the feature map. In the semantic segmentation network, the larger the receptive field of the final predicted pixels, the better segmentation performance the deep network can achieve. In addition, it should be mentioned that the use of dilated convolution does not increase the number of parameters and computations.

**Figure 4:**
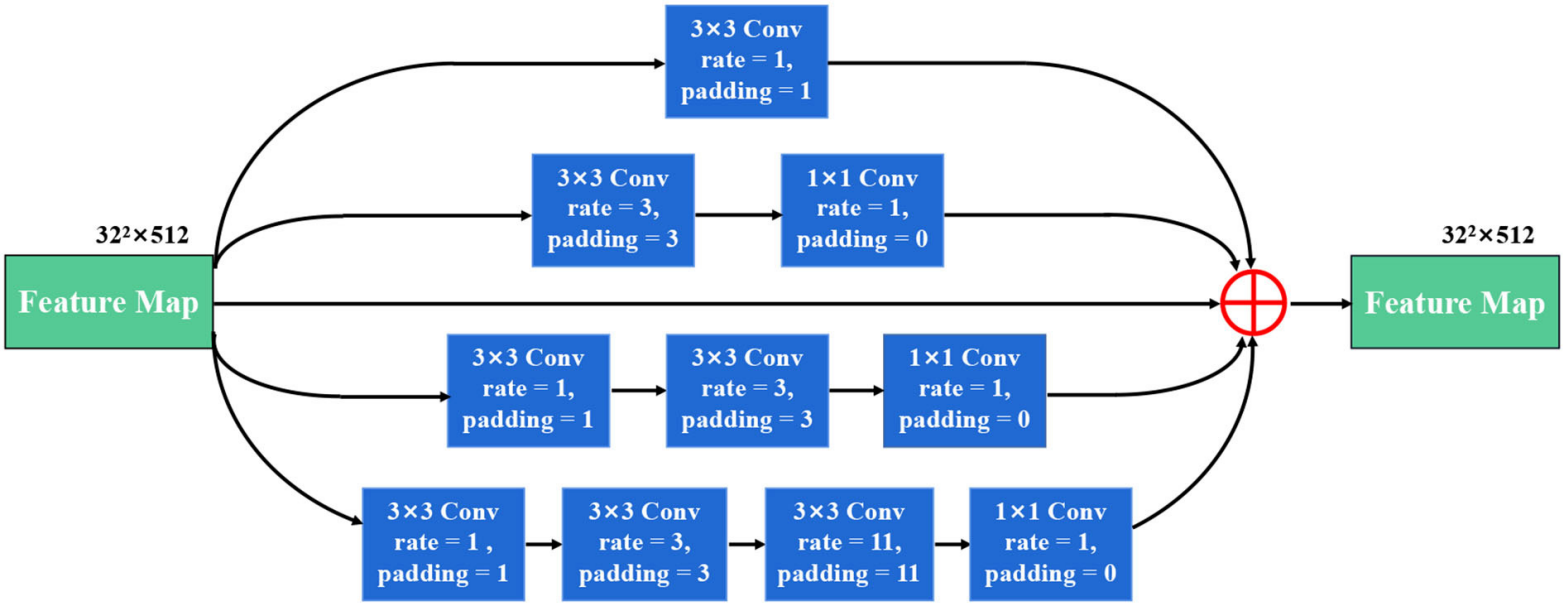
The illustration of the stackable dilated convolution (SDC) block. It has four branches of dilated convolution stack: 3x3 convolutional layer. Each branch is stacked with dilated convolutions with expansion rates of 1, 3, and 11. The SDC block can extract features from different scales.

As shown in Figure 4, our SDC block has four branches. The four-branch structure is utilized since this is a tradeoff between accuracy and computation burden. Each branch in SDC is stacked with dilated convolutional layers with different dilation rates. As a result, the SDC has four branches of dilated convolution stack. The size of the convolution kernel is 3 × 3, and the expansion coefficient will gradually increase, from 1 to 1, 3, and 11. Thus, the receptive field of each branch will be 3, 7, 9, and 31, respectively. It employs different receptive fields, similar to Inception (Szegedy et al., 2017) structures. In order to keep the number of channels constant, we set the same padding parameter as the dilation rates. At the end of each dilated branch, we apply a 1 × 1 convolutional layer for corrected linear activation. Finally, we directly add the original features with other features *via* a skip connection. In general, the convolution of a large reception field can extract and generate abstract features for large objects, while the convolution of a small reception field is good at segmenting small objects. By stacking the dilated convolution with different dilation rates, the SDC block is able to allow the network to learn contextual information and extract features for objects of various sizes. The four cascaded dilated convolution branches are added to the feature map itself and then sent to the subsequent decoding module. The input size is the same as the output size in the SDC module.

## 4. Experiment results

### 4.1. Experimental setup

#### 4.1.1. Two datasets

The TNUI-2021 dataset was used to evaluate our N-Net, which consists of 1,381 ultrasound thyroid nodule images. The resolution of each image is 780 × 780. This dataset was acquired from 483 patients by doctors in the Fujian Medical University Union Hospital using two apparatuses, Supersonic Aixplorer and SAMSUNG WS80A. In the TNUI-2021 dataset, each image is paired with ground truth (GT) image. The GT images were manually labeled by the expert pathologists in the Fujian Medical University Union Hospital. The labels were stored in tabular files, and benign and malignant classifications were also labeled. There are 72 images of benign nodules and 1,309 images of malignant nodules.

The DDTI dataset contains 637 ultrasound thyroid nodule images of different resolution sizes, such as 560 × 360, 280 × 360, and 245 × 360. The ratio of the training set, validation set, and test set is 6:2:2, and the augmented patches are resized to the size of 512 × 512 for training.

#### 4.1.2. Experiment settings

Considering the serious imbalance of these two types of data, we aggregated the data of benign and malignant nodules to divide the data set. We employed a 5-fold cross-validation method to evaluate the performance of our N-net. Specifically, the TNUI-2021 dataset was randomly and equally divided into five non-overlapping sub-datasets. For each time, 20% of images were used for testing, and the remaining 80% of images were used for training and validation. The ratio of the training set, validation set, and test set in each experiment was 6:2:2. The averaged results of five validations were obtained as the final results. The N-Net training and testing were completed in PyTorch. The training and testing platform was the Ubuntu 18.04 system with an NVIDIA GeForce RTX 2070 graphics card, which has 8 Gigabyte memory.

#### 4.1.3. Training phase

In our N-Net, in order to reduce the risk of overfitting, we enlarged the training dataset with the online data augmentation processing, including rotation of images at 90, 180, and 270-degree angles, horizontal flip, and vertical flip. Then we resized the augmented patches to the size of 512 × 512 for training. The Adam optimizer (Kingma and Ba, 2014) with a batch of 16 and 32 was used to optimize the segmentation task. We set the initialized learning rate to 0.0001. To avoid our network trapping in overfitting during training, we employed the validation datasets to supervise them and terminated the training process after 300 epochs.

#### 4.1.4. Testing phase

To improve the robustness of medical image segmentation method, we also adopted an augmentation strategy on testing dataset, as that in Dai et al. (2016); Zhang et al. (2018), including image rotation of images at 90, 180, and 270 degree angles, horizontal flip, and vertical flip (equal to predicting each image 6 times). In the end, we used the average of six prediction maps to get the final prediction map. All baseline approaches utilized the same strategy during the testing phase.

### 4.2. Evaluation metrics

To evaluate the segmentation performance, several metrics were used in our experiments, including dice coefficient (Dice), mean intersection over union (mIoU), Precision, Recall, and F1-Score, which were calculated as follows:


(1) $$\begin{array}{r} Dice=\frac{2TP}{FP+2TP+FN}, \end{array}$$

(2) $$\begin{array}{r} mIoU=\frac{1}{k+1}\overset{k}{\underset{i=0}{\sum }}\frac{TP}{FP+TP+FN}, \end{array}$$

(3) $$\begin{array}{r} Precision=\frac{TP}{TP+FP}, \end{array}$$

(4) $$\begin{array}{r} Recall=\frac{TP}{TP+FN}, \end{array}$$

(5) $$\begin{array}{r} F1-Score=2\frac{RecallPrecision}{Precision+Recall}, \end{array}$$

where TP, FP, and FN denote the number of true positives, false positives, and false negatives, respectively. We used the Dice, mIoU, Precision, Recall, and F1-Score to measure the segmentation performance.

### 4.3. Comparisons with different segmentation methods

**Figure 5:**
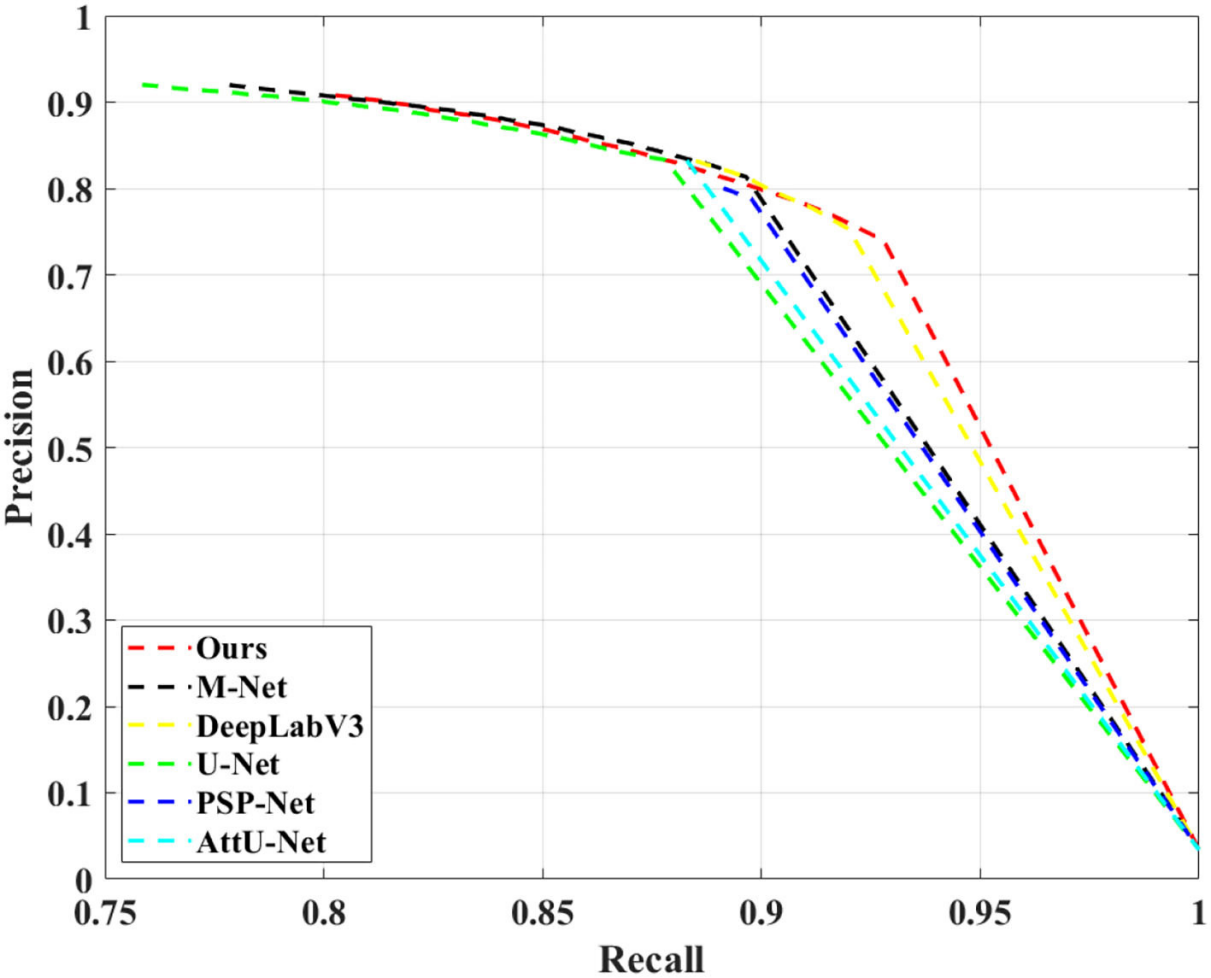
Comparison of precision-recall curves of our N-Net and other medical image segmentation approaches on the TNUI-2021 dataset.

**Figure 6:**
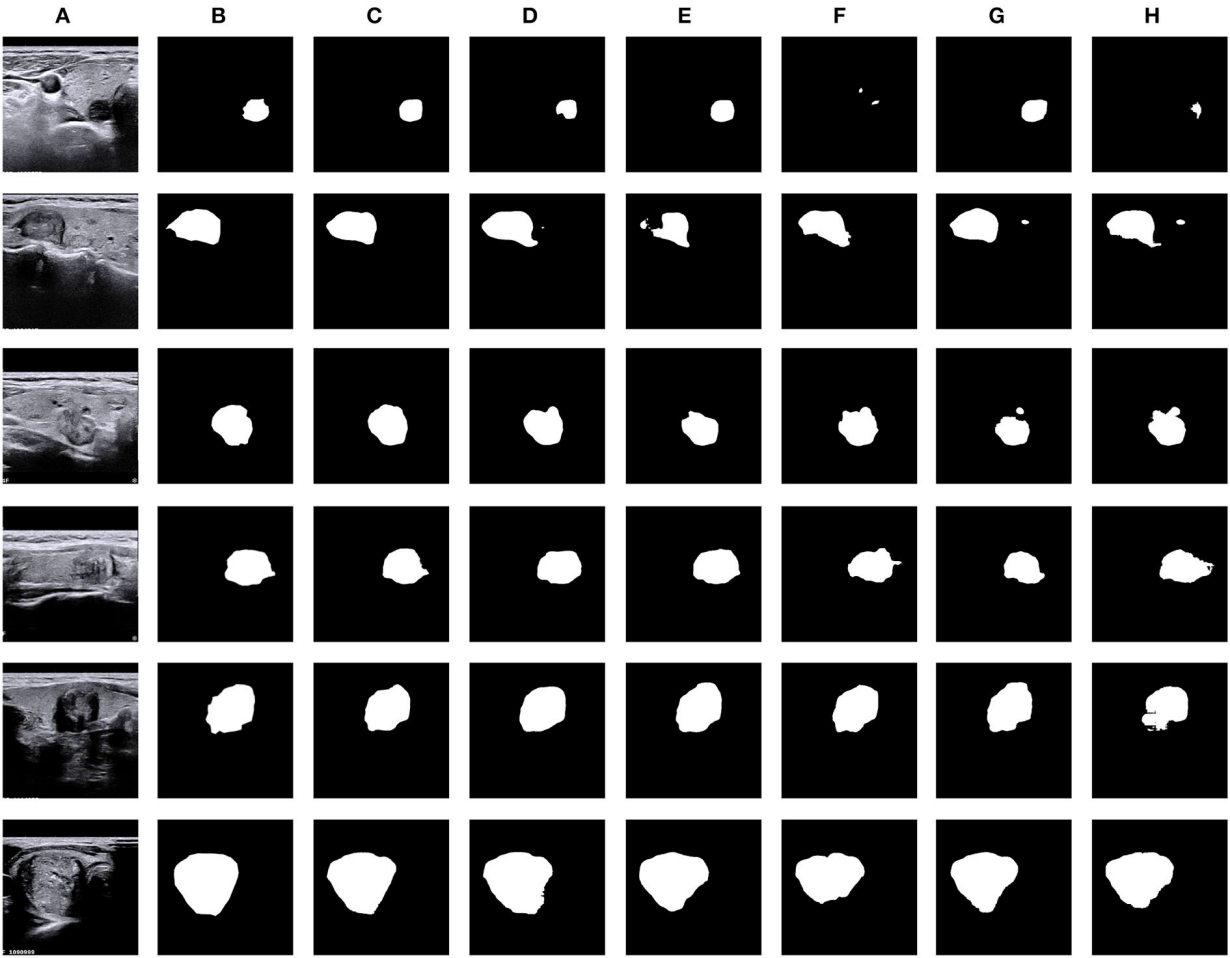
Visual comparison of thyroid nodule segmentation results generated from five typical methods, including our N-Net. **(A)** Input, **(B)** GT, **(C)** Ours, **(D)** DeepLabV3, **(E)** M-Net, **(F)** PSP-Net, **(G)** AttU-Net, and **(H)** U-Net.

We compared the proposed N-Net model with several different segmentation approaches in Table 1, including a U-shape convolutional network (U-Net) (Ronneberger et al., 2015), an attention U-Net (AttU-Net) (Oktay et al., 2018), a pyramid scene parsing network (PSP-Net) (Zhao et al., 2017), a Unet++ network (Zhou et al., 2019), a multi-label deep network (M-Net) (Fu et al., 2018), and an atrous convolution for semantic image segmentation network (DeepLabV3) (Chen et al., 2017a). Five metrics were used, including Dice, mIoU, Precision, Recall, and F1-Score to evaluate the segmentation performance of those methods. A quantitative comparison is shown in Table 1. Moreover, we have drawn the Precision-Recall curve to compare our N-Net with other medical image segmentation approaches, as shown in Figure 5. As can be seen, our N-Net model achieves the best performance on the TNUI-2021 dataset. For the segmentation task of thyroid nodule ultrasound images, our model achieves 0.9195, 0.8721, 0.8888, 0.8326, and 0.8437 in Dice, mIoU, Precision, Recall, and F1-Score, more accurate than other methods. Compared with the backbone U-Net (Ronneberger et al., 2015), the Dice increases from 0.8887 to 0.9195 by 3.08%, the mIoU increases from 0.8372 to 0.8721 by 3.49%, the Precision increases from 0.8506 to 0.8888 by 3.82%, the Recall increases from 0.7731 to 0.8326 by 5.95%, the F1-Score increases from 0.784 to 0.8437 by 5.97%, which demonstrates that the proposed N-Net architecture is beneficial for thyroid nodule ultrasound images segmentation. The segmentation results using different methods are visually compared in Figure 6. As we can see from the updated result in Table 1 when the cross-validation was adopted, the performance of our N-Net is significantly better than that of the DeepLabV3. The quantitative comparison in the DDTI dataset is shown in Table 2. It can be noted that our N-Net has a gap compared with the first place in the TN-SCUI2020 network (Wang et al., 2020). Due to a large number of model parameters, the average time to predict each ultrasound image will increase, which is unfavored by clinicians in diagnosis in real time. Our method was utilized since this is a trade-off between accuracy and computation burden. We could achieve a good segment accuracy by using a one-stage network with fewer parameters, and it can make the diagnosis in a shorter time.

**Table 1:** Thyroid nodule segmentation performance of different segmentation approaches on the TNUI-2021 dataset.

**Method**	**Dice (%)**	**mIoU (%)**	**Precision (%)**	**Recall (%)**	**F1-Score (%)**
U-Net (Ronneberger et al., 2015)	88.87 ± 0.62	83.72 ± 0.55	85.06 ± 1.66	77.31 ± 0.72	78.40 ± 1.19
AttU-Net (Oktay et al., 2018)	89.45 ± 0.65	84.42 ± 0.56	84.87 ± 0.82	79.13 ± 2.32	79.53 ± 1.27
PSP-Net (Zhao et al., 2017)	89.25 ± 0.38	83.91 ± 0.51	85.37 ± 1.81	78.34 ± 2.53	79.12 ± 0.74
U-Net++ (Zhou et al., 2019)	91.41 ± 0.48	86.86 ± 0.44	87.01 ± 0.91	82.91 ± 1.07	83.68 ± 0.90
M-Net (Fu et al., 2018)	91.52 ± 0.57	86.67 ± 0.51	88.06 ± 1.37	82.36 ± 1.75	83.6 ± 1.12
DeepLabV3 (Chen et al., 2017a)	91.66 ± 0.6	86.88 ± 0.67	88.04 ± 0.93	82.97 ± 2.11	83.81 ± 1.13
Ours	**91.95** **±0.12**	**87.21** **±0.22**	**88.88** **±1.29**	**83.26** **±1.17**	**84.37** **±0.18**

The best results are in bold. The average of 5-fold cross-validation ± SD.

**Table 2:** Thyroid nodule segmentation performance of different segmentation approaches on the DDTI dataset.

**Method**	**Dice (%)**	**mIoU (%)**	**Precision (%)**	**Recall (%)**	**F1-Score (%)**	**Flops (G)**	**Time (ms)**
U-Net (Ronneberger et al., 2015)	84.17	76.29	70.48	81.23	71.57	46.42	27.75
AttU-Net (Oktay et al., 2018)	84.91	77.37	71.37	81.7	72.76	266.47	84.81
PSP-Net (Zhao et al., 2017)	81.25	73.36	69.08	73.72	65.91	**9.43**	**4.96**
M-Net (Fu et al., 2018)	86.4	79.38	80.45	76.59	75.45	63.59	31.58
DeepLabV3 (Chen et al., 2017a)	87.72	82.66	82.77	82.88	79.54	109.33	48.69
nnU-Net (Isensee et al., 2021)	88.59	80.76	82.27	85.23	82.79		
Ours	88.76	82.69	81.53	82.94	79.62	68.52	36.96
1st in TN-SCUI2020 (Wang et al., 2020)	92.39	86.84	84.33	86.71	86.37	410.58	108.49
Two-stage cascaded of Ours	**93.67**	**88.46**	**90.4**	**91.94**	**90.62**	137.04	73.92

The best results are shown in bold.

**Figure 7:**
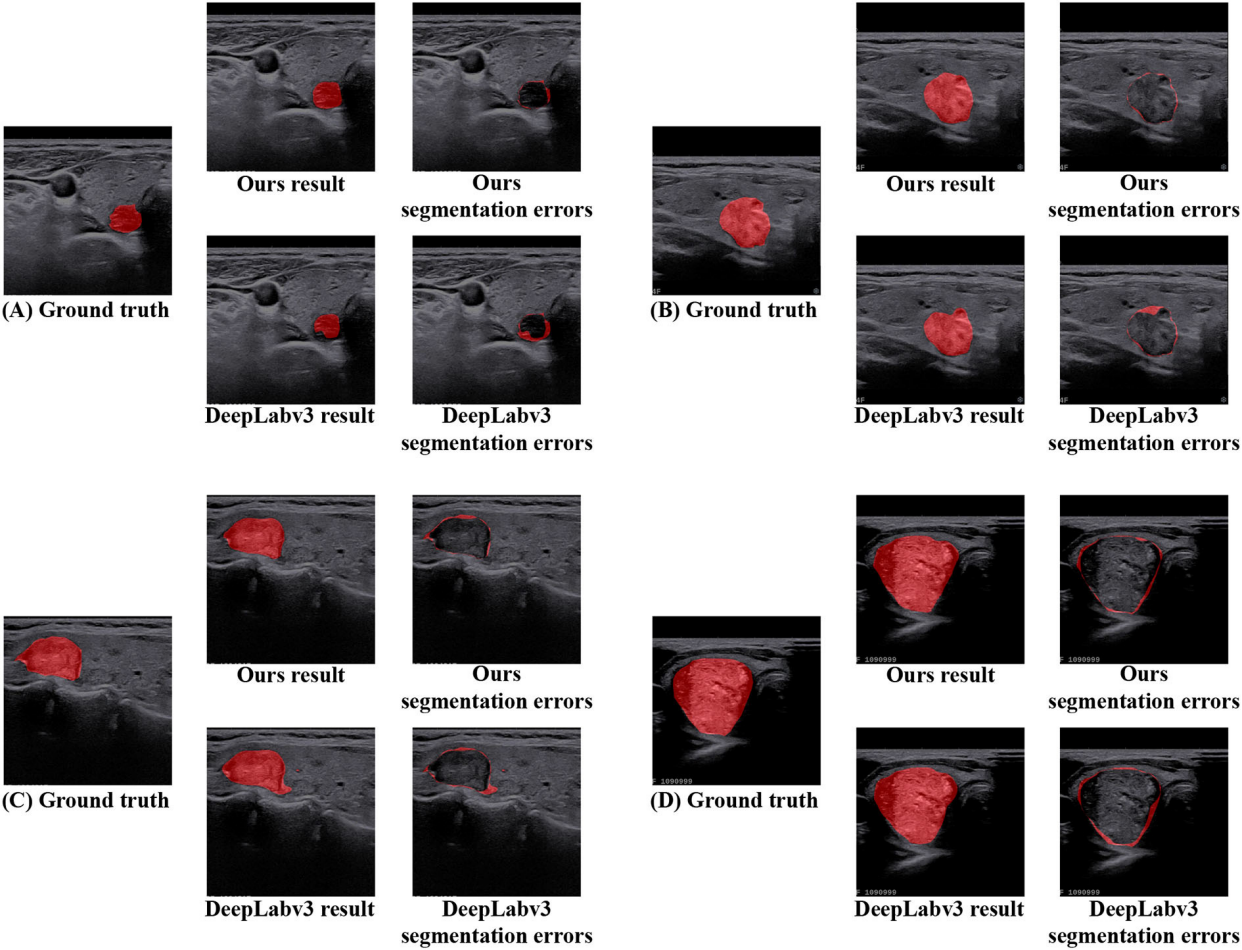
The segmentation area and error of DeepLabV3 and our N-Net methods are further visualized on the TNUI-2021 dataset. **(A–D)** Ground truth, ours result, ours segmentation errors, DeepLabv3 result, and DeepLabv3 segmentation errors.

In addition, in terms of visualized segmentations as shown in Figures 6, 7, we can find that our network achieves better segmentation results at the edge of the target region than that of the DeepLabV3 (Chen et al., 2017a). Since the DeepLabV3 network uses direct bilinear upsampling 16 times to obtain the segmentation result, the boundary and detail information has been lost in the feature extraction process, which may not be fully restored during decoding. In our network, the detailed information obtained by the encoder is propagated to the subsequent decoder through skip connections, thereby effectively solving the loss of edge information caused by sampling.

### 4.4. Ablation study

**Figure 8:**
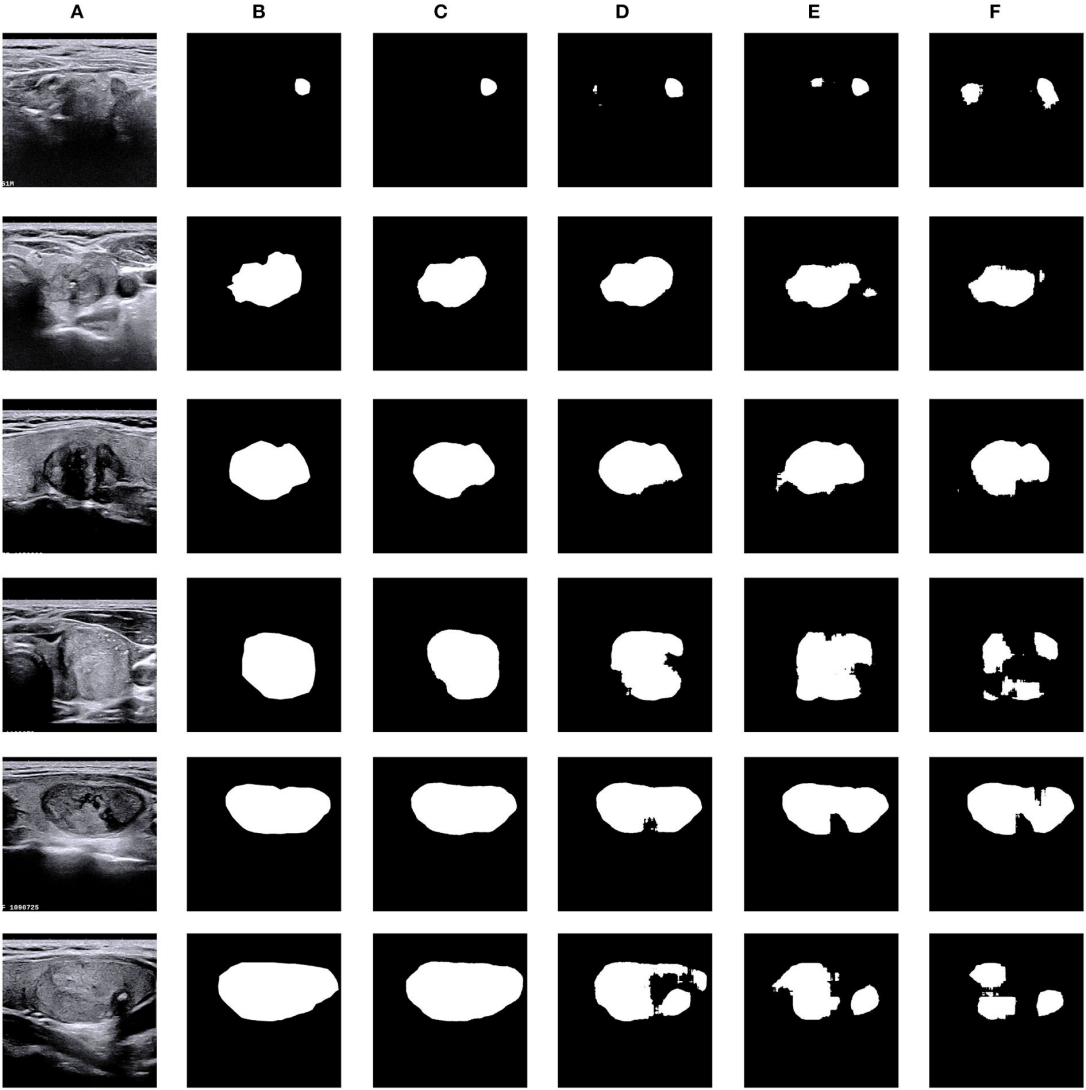
Visual comparison of ablation study on the TNUI-2021 dataset. **(A)** Input, **(B)** GT, **(C)** ours, **(D)** multi-scale+AG +U-Net, **(E)** AG+U-Net, and **(F)** U-Net.

Ablation studies were performed to analyze the effectiveness and the contributions of each module in the proposed N-Net model on the TNUI-2021 dataset. In addition, in terms of visualized segmentations, the results are shown in Figure 8.

#### 4.4.1. Effect of the attention guidance module

Our proposed N-Net model employs the U-Net (Ronneberger et al., 2015) as a baseline model. We used the attention guidance module to guide the learning of more accurate semantic features of target regions during downsampling by channel fusion of the input images and the feature maps after convolutional layers, thus providing rich information for improving segmentation. Specifically, this module was used to highlight the thyroid nodule region and to reduce the background effects. Table 3 shows how the quantitative performance changes by adding an attention guidance module to an improved U-Net (Ronneberger et al., 2015) architecture. As we can see, the Dice, mIoU, Recall, and F1-Score significantly increase with the attention guidance module compared to without: the Dice is increased by 0.49% from 0.8887 to 0.8936, the mIoU is increased by 0.52% from 0.8372 to 0.8424, the Recall is increased by 2.51% from 0.7731 to 0.7982, and the F1-Score is increased by 0.95% from 0.7840 to 0.7935, respectively, with the exception that the Precision is decreased by 0.07% from 0.8506 to 0.8499. The above results indicate that the attention guidance module is beneficial for our segmentation task.

**Table 3:** Ablation study for each component of our model for thyroid nodule segmentation on the TNUI-2021 dataset. The average of 5-fold cross-validation ± SD.

**Method**	**Dice (%)**	**mIoU (%)**	**Precision (%)**	**Recall (%)**	**F1-Score (%)**
Backbone (U-Net)	88.87 ± 0.62	83.72 ± 0.55	85.06 ± 1.66	77.31 ± 0.72	78.40 ± 1.19
Attention guidance (AG) + U-Net	89.36 ± 0.42	84.24 ± 0.4	84.99 ± 1.05	79.82 ± 1.33	79.35 ± 0.83
Multi-scale + AG + U-Net	90.28 ± 0.46	85.34 ± 0.55	85.75 ± 1.61	80.99 ± 2.52	81.13 ± 0.92
SDC + Multi-scale + AG + U-Net	**91.95** **±0.12**	**87.21** **±0.22**	**88.88** **±1.29**	**83.26** **±1.17**	**84.37** **±0.18**

The bold values indicates the best results.

#### 4.4.2. Effect of adopting multi-scale input layer

Table 3 also shows the effect of the multi-scale input layer, which further improves the performance of thyroid nodule ultrasound image segmentation. Compared to the “Attention guidance (AG) module + U-Net,” the Dice increases from 0.8936 to 0.9028 by 0.92%, the mIoU increases from 0.8424 to 0.8534 by 1.1%, the Precision increases from 0.8499 to 0.8575 by 0.76%, the Recall increases from 0.7982 to 0.8099 by 1.17%, and the F1-Score increases from 0.7935 to 0.8113 by 1.78%, respectively. We improved the multi-scale input layer by integrating the attention guidance module and SDC block. The multi-scale input layer builds an image pyramid to provide both local and global information at different scales into the network and extract multi-scale features for fusion, which greatly improves the performance of the entire network. Thus, the above results demonstrate that the multi-scale input layer is effective in our N-Net model.

#### 4.4.3. Effect of the stackable dilated convolution block

The SDC block we proposed has four branches similar to Inception structures, and each branch is stacked with dilated convolutions with different dilation rates. One advantage of the SDC block is that during feature extraction, the size of the receptive field completely covers a square area without any holes or missing edges. This means that it can avoid the gridding effects when using dilated convolution, allowing the deep network to learn more accurate semantic features and improve the accuracy of the model segmentation task. Another benefit of the SDC block is that it can be achieved by stacking dilated convolutions with different dilation rates, thus naturally enlarging the receptive fields of the network without adding more modules. This is important for segmenting relatively large target regions. In addition, the SDC block is naturally integrated with the original layers of the network, without adding extra blocks. We set the input and output feature maps of the SDC block to have the same size and channels.

As shown in Table 3, our proposed SDC module improves the Dice, mIoU, Precision Recall, and F1-Score in thyroid nodule ultrasound image segmentation. Compared to the 'Multi-scale input layer + Attention guidance (AG) module + U-Net', the Dice increases from 0.9028 to 0.9195 by 1.67%, the mIoU increases from 0.8534 to 0.8721 by 1.87%, the Precision increases from 0.8575 to 0.8888 by 3.13%, the Recall increases from 0.8099 to 0.8326 by 2.27%, and the F1-Score increases from 0.8113 to 0.8437 by 3.24%, respectively. It reveals that our SDC block is useful for the segmentation task, and it may help to extract more high-level semantic information compared to the standard convolution.

## 5. Conclusion

In this study, we propose a relatively new N-Net for thyroid nodule segmentation. First, we proposed to use a multi-scale input layer to achieve multi-level receiver field sizes and obtain rich feature representations. After that, the U-shape convolutional network was employed as the backbone structure. Moreover, we proposed an attention guidance module to filter the features before several skip connections, which can transfer structural information from the previous feature maps to the following layers. This module can also remove noise and reduce the negative impact of the background. Finally, we propose a stackable dilated convolution (SDC) block, which is able to capture and encode deeper semantic features that may be lost in bilinear upsampling. Experimental results on the TNUI-2021 dataset and the DDTI dataset demonstrate that the proposed N-Net model outperforms several typical segmentation approaches.

## Data availability statement

The raw data supporting the conclusions of this article will be made available by the authors, without undue reservation.

## Ethics statement

The studies involving human participants were reviewed and approved by Ethics Committee of Fujian Medical University Union Hospital. The patients/participants provided their written informed consent to participate in this study. Written informed consent was obtained from the individual(s) for the publication of any potentially identifiable images or data included in this article.

## Author contributions

XN, XZ, TT, XL, LW, HZ, JL, SC, and MD: concept and design. XN, XZ, HZ, EX, SC, MZ, CC, and TT: acquisition of data. XN, XZ, TT, XL, LW, and SC: model design and manuscript drafting. XN, XZ, XL, LW, and TT: data analysis. XN, XZ, TT, XL, LW, HZ, JL, EX, SC, MZ, CC, and MD: approval. All authors contributed to the article and approved the submitted version.

## Funding

This study was supported by the National Natural Science Foundation of China under (Grant Nos. 61901120 and 62171133), sponsored by Fujian Provincial Health Technology Project (2019-1-33), in part by the Science and Technology Innovation Joint Fund Program of Fujian Province of China under (Grant No. 2019Y9104), and the Guiding Projects of Fujian Provincial Technology Research and Development Program under (Grant No. 2022Y0023).

## Conflict of interest

Author TT was employed by Imperial Vision Technology. The remaining authors declare that the research was conducted in the absence of any commercial or financial relationships that could be construed as a potential conflict of interest.

## Publisher's note

All claims expressed in this article are solely those of the authors and do not necessarily represent those of their affiliated organizations, or those of the publisher, the editors and the reviewers. Any product that may be evaluated in this article, or claim that may be made by its manufacturer, is not guaranteed or endorsed by the publisher.
