# Corrigendum: N-Net: A novel dense fully convolutional neural network for thyroid nodule segmentation

**DOI:** 10.3389/fnins.2022.1034239

**Published:** 2022-09-20

**Authors:** Xingqing Nie, Xiaogen Zhou, Tong Tong, Xingtao Lin, Luoyan Wang, Haonan Zheng, Jing Li, Ensheng Xue, Shun Chen, Meijuan Zheng, Cong Chen, Min Du

**Affiliations:** ^1^College of Physics and Information Engineering, Fuzhou University, Fuzhou, China; ^2^Fujian Key Lab of Medical Instrumentation and Pharmaceutical Technology, Fuzhou University, Fuzhou, China; ^3^Imperial Vision Technology, Fuzhou, China; ^4^Fujian Medical Ultrasound Research Institute, Fuzhou, China; ^5^Department of Ultrasound, Fujian Medical University Union Hospital, Fuzhou, China

**Keywords:** deep convolutional neural network, medical image segmentation, dilated convolution, multi-scale input layer, thyroid nodule

In the published article, there was an error regarding the affiliations for authors Ensheng Xue, Shun Chen, Meijuan Zheng, Cong Chen. As well as having affiliation(s) 4 they should also be affiliated to “5 Department of Ultrasound, Fujian Medical University Union Hospital, Fuzhou, China.”

The authors apologize for this error and state that this does not change the scientific conclusions of the article in any way. The original article has been updated.

## Publisher's note

All claims expressed in this article are solely those of the authors and do not necessarily represent those of their affiliated organizations, or those of the publisher, the editors and the reviewers. Any product that may be evaluated in this article, or claim that may be made by its manufacturer, is not guaranteed or endorsed by the publisher.

